# Guanine is an inhibitor of c-jun terminal kinases

**DOI:** 10.1038/s41598-025-11617-3

**Published:** 2025-08-11

**Authors:** Jessica Treeby, Sherihan El-Sayed, Samuel Morgan, Sophie Maddock, George Taylor, Stacey Warwood, Julian Selley, David Knight, Benjamin Saer, Richard A. Bryce, Jean-Michel Fustin

**Affiliations:** 1 Faculty of Biology, Medicine and Health, Centre for Biological Timing, The University of Manchester, Manchester, UK; 2 Division of Pharmacy and Optometry, School of Health Sciences, Manchester Academic Health Sciences Centre, University of Manchester, Oxford Road, Manchester, M13 9PT UK; 3 Department of Medicinal Chemistry, Faculty of Pharmacy, Zagazig University, Zagazig, 44519 Egypt; 4 FBMH Platform Sciences, The University of Manchester, Enabling Technologies & Infrastructure, BioMS, Manchester, UK

**Keywords:** Guanine, Purine, JNK, MAPK, Lesch-Nyhan, Metabolism, Biochemistry, Kinases, Metabolic disorders, Cell biology, Circadian rhythms, Mechanisms of disease

## Abstract

The toxicity of purine bases adenine and guanine is mostly recognized when associated with inborn errors of purine metabolism such as Lesch-Nyhan syndrome, and metabolic diseases with a lifestyle component including gout. In these pathologies, the peripheral toxicity of purine bases is attributed to the accumulation of their catabolite uric acid in the kidneys, causing nephrolithiasis or crystalluria, and the joints, causing gout. However, inborn errors of purine metabolism also present neurological and neurobehavioral abnormalities including motor disabilities, seizures, hypotonia and dystonia, and self-injurious behaviour. The mechanisms underlying these pathologies is less well-understood but does not seem to be caused by uric acid. In a different context, adenine and guanine have been shown to be cytotoxic and antiproliferative, highlighting their potential use in cancer chemotherapies, but the underlying mechanisms have not been identified. In our previous investigations, we have shown that adenine, a molecule classified as acutely toxic, is an inhibitor of 1-carbon metabolism and biological methylations. Using the same experimental paradigm based on real-time luminometry with mouse embryonic fibroblasts to probe in real-time the potential biological activity of small molecules, complemented with metabolite quantifications, in silico docking predictions, kinase assays and phosphoproteomics, we now reveal that guanine and to a lesser extent adenine are direct inhibitors of c-Jun N-terminal kinases, which may contribute to their toxicity and to the symptoms of Lesch-Nyhan syndrome.

**Supplementary Information:**

The online version contains supplementary material available at 10.1038/s41598-025-11617-3.

## Introduction

 The purine nucleotides ATP and GTP are not only components of nucleic acid DNA and RNA but are also required for many essential processes including energy metabolism and signal transduction. Moreover, the purine nucleoside adenosine is a neurotransmitter^1^ and guanosine a neuromodulator^2^. Therefore, it is not surprising that the metabolism of purine is tightly regulated. The purine nucleotides GMP and AMP can either be synthesized *de novo* from inosine monophosophate (IMP) or salvaged from their respective nucleosides (guanosine and adenosine) and bases (guanine and adenine). The degradation of all purines converges in the production of the base xanthine, then uric acid.

Considering their importance for cellular metabolism, it is not surprising that disorders of purine metabolism, genetic and life-style related, lead to dramatic pathologies. A chronic dietary excess in purines causes gout due to the accumulation of uric acid in the joints. Similarly, genetic mutations inactivating the enzyme hypoxanthine-guanine phosphoribosyltransferase (HPRT) that salvages guanine to GMP leads to increased degradation of guanine to uric acid, causing gout^3,4^. Lesch-Nyhan disease is caused by mutations in HPRT, and while gout may not arise or be detected until late childhood, cognitive impairment, motor disability and self-injurious behaviour including biting of the lips and fingers, head-banging and poking of the eyes occur much earlier^3,5^. The mechanisms underlying such neurological symptoms are not well understood but do not seem to involve neurodegeneration or abnormal morphology^6,7^. A disruption in the development of the dopaminergic and adrenergic pathway has been proposed, but little evidence of the underlying cause has been provided^8,9^.

Lesch-Nyhan disease highlights the potential toxicity of purine bases, but the lack of evidence for the mechanisms underlying the toxicity of purines limits treatment opportunities. Adenine and guanine have been shown to be intrinsically cytotoxic in several cell types in vitro, a toxicity that appeared to not depend on the formation of secondary purine metabolites^10–13^. In vitro approaches have provided other important clues: cultures of Lesch-Nyhan fibroblasts have established that the severity of the disease is strongly correlated with the inability to salvage guanine by HPRT^14^and HPRT knock-out cell lines have shown that guanine, undetectable in wild-type control cells, considerably increased, associated with slower proliferation rates^15^.

Investigating the mechanisms underlying purine toxicity, we have previously demonstrated that adenine is an inhibitor of 1-carbon metabolism and cellular methylations^16^. Here, using real-time luminometry of cellular circadian rhythms as a toxicity screening, in silico docking predictions, in vitro kinase assays and phosphoproteomics, we report that guanine is an inhibitor of the c-Jun N-terminal kinases JNK1-3, with an IC50 of 100–300 µM.

## Results

To investigate the biological activity and adverse effects of the purine bases adenine, guanine, xanthine and hypoxanthine, we used a real-time luminescence assay with mouse embryonic fibroblasts prepared from PER2::LUC mice carrying a heterozygous chimeric fusion between the endogenous circadian clock protein PER2 and LUCIFERASE^17^. Since the circadian clock is based on a transcription-translation feedback loops driving rhythmic gene expression, and controlled by cellular metabolism at all levels from epigenetic to post-translational regulations, this system is particularly well suited to detect and quantify the adverse effects of small molecules and inhibitors, as we have demonstrated previously with adenine and other 1-carbon metabolism inhibitors^16,18–20^.

**Fig. 1:**
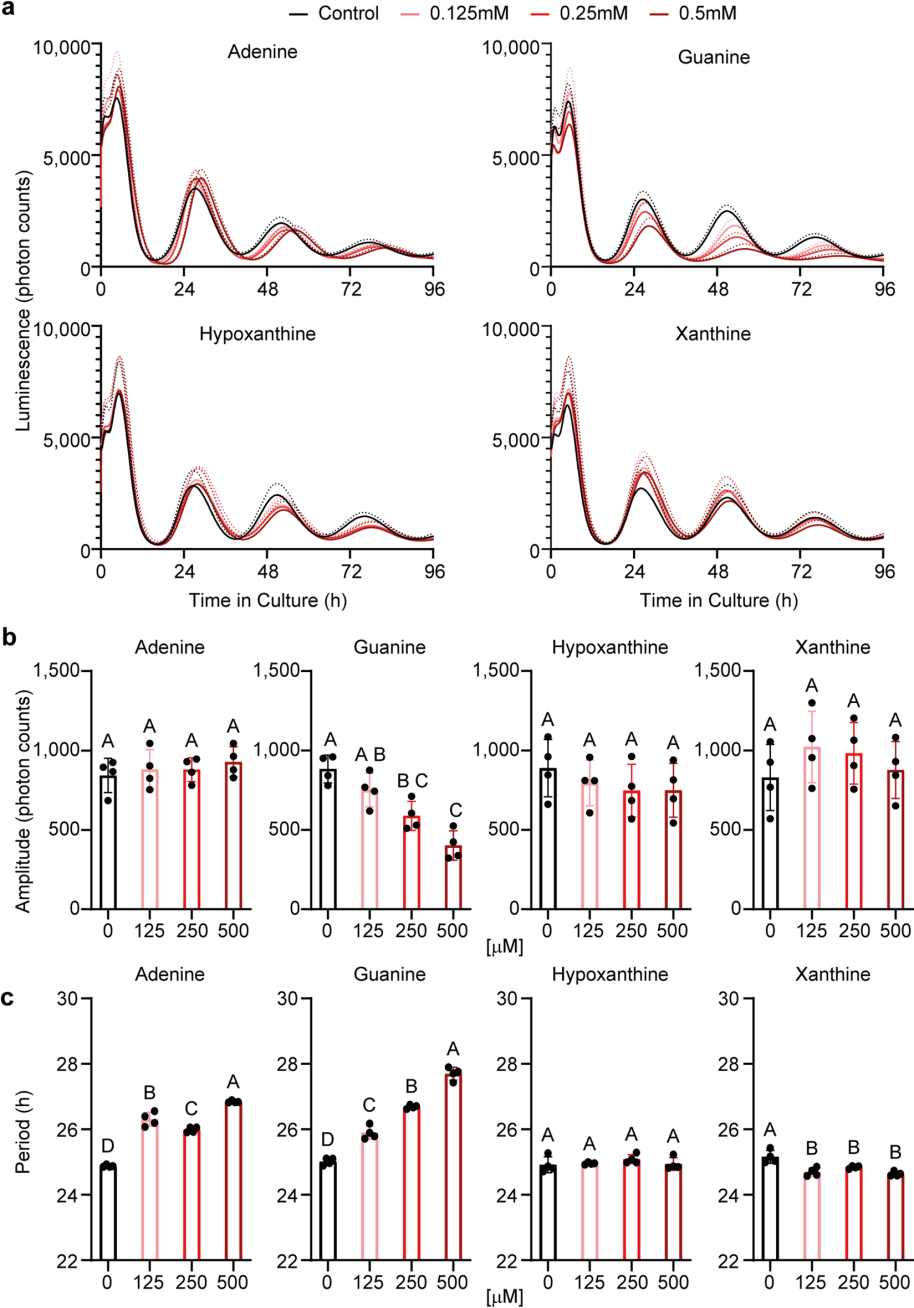
Guanine lengthen the circadian period and decrease the amplitude of oscillations**. a**, Dexamethasone-synchronized PER2::LUC MEFs were treated with either adenine, guanine, xanthine or hypoxanthine at the concentrations indicated above and luminescence was acquired in real-time for at least 96 h (representative results of at least 3 independent experiments, data showing mean of *n* = 4 independent replicate dishes with + SD shown as a dotted line for each trace). From these traces, amplitude (**b**) and period (**c**) were estimated by Biodare2. Data show mean +/- SD of *n* = 4 independent replicate dishes, analysed by one-way ANOVA followed by Bonferroni’s test; within each graph, letters above bars indicate significance (at least *P* < 0.05) in Bonferroni’s comparisons, e.g. A vs. B or AB vs. C are significant but A vs. A or vs. AB are not.

As previously observed^16^adenine caused a concentration-dependent lengthening of the circadian period, i.e. a slower biological clock (Fig. 1a, b). Guanine was even more potent than adenine, not only causing a lengthening of the circadian period (Fig. 1a, b) but also accompanied with a decrease in amplitude (Fig. 1c), which is a measure of the robustness of circadian oscillations. Xanthine showed a weak shortening of the period, but no concentration-dependent effects were seen (Fig. 1a, b). Hypoxanthine did not affect the oscillations, the period or the amplitude of reporter gene oscillations. We further investigated the mechanisms underlying the effects of guanine.

**Fig. 2:**
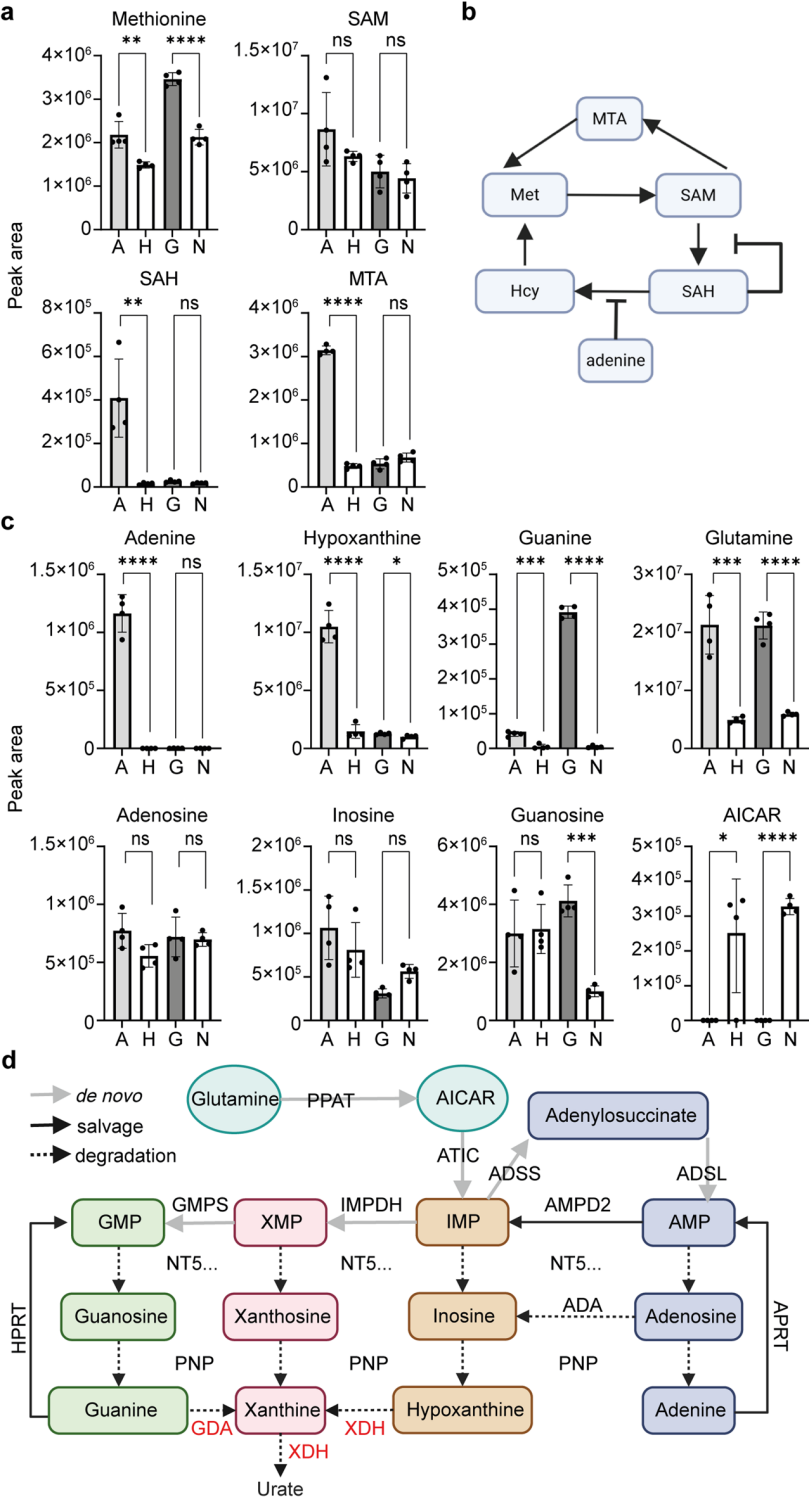
Metabolic response to excess adenine and guanine.** a**, Quantification of selected 1-carbon metabolites by HPLC in cells treated with adenine (A), guanine (G) or their respective vehicle (H, HCl; N, NaOH), data show mean of n = 4 independent replicate dishes with +/-SD analysed by unpaired t test. **b**, Simplified 1-carbon metabolism, with adenine an inhibitor of SAH hydrolysis by AHCY and SAH an inhibitor of methyltransferases using SAM. **c**, Quantification of purine bases and nucleosides by HPLC in cells treated with adenine, guanine or their respective vehicle (H, HCl; N, NaOH), data show mean of n = 4 independent replicate dishes with +/-SD analysed by unpaired t test. **d**, Simplified purine metabolism, showing the name of enzymes that have been detected in PER2::LUC cells in our proteome dataset (see below) and RNASeq data previously published^16^. Guanine deaminase (GDA) and Xanthine dehydrogenase (XDH), essential for guanine and xanthine/hypoxanthine degradation, were not detected. Other abbreviations: PPAT, phosphoribosyl pyrophosphate amidotransferase; ATIC, 5-aminoimidazole-4-carboxamide ribonucleotide formyltransferase/IMP cyclohydrolase; ADSS, Adenylosuccinate synthase; ADSL, Adenylosuccinate lyase; ADA, Adenosine deaminase; IMPDH, inosine monophosphate dehydrogenase; GMPS, guanosine monophosphate synthase; AMPD, adenosine monophosphate deaminase; NT5., 5’-nucleotidase (many homologues present); APRT, adenine phosphoribosyltransferase; PNP, purine nucleoside phosphorylase.

Since adenine was shown to disrupt the methyl cycle by inhibiting the enzyme adenosylhomocysteinase (AHCY), leading to changes in 1-carbon metabolites adenosylmethionine (SAM), adenosylhomocysteine (SAH) and methylthioadenosine (MTA)^16^we first hypothesized that guanine had the same mode of action. To investigate this possibility and to gain insights into the metabolic paths of exogenous guanine and adenine, we quantified selected 1-carbon metabolites in cells treated with these 2 purine bases. Adenine caused an increase in SAH (Fig. 2a), confirming the inhibition of AHCY previously observed^16^accompanied by an increase in methionine and MTA, suggesting the methionine salvage pathway has been activated. In contrast, guanine had no effect on SAH or MTA (Fig. 2a), evidence that guanine does not inhibit AHCY. A graphical representation of simplified 1-carbon metabolism is shown in Fig. 2b to guide understanding.

To gain further insights into how guanine is metabolized in these cells and whether guanine treatment leads to intracellular guanine accumulation, we quantified purine bases and nucleosides (Fig. 2c). In cells treated with adenine, we observed an increase not only in adenine but also in hypoxanthine and guanine, indicating excess adenine is indirectly converted to guanine and catabolized to hypoxanthine, and/or allosterically stimulates the *de novo* branch to guanine nucleotides. In contrast, guanine but not adenine increased in cells treated with guanine, indicating there is no conversion of guanine to adenine in these cells. In cells treated with adenine or guanine, AICAR dramatically dropped while glutamine increased, confirming that *de novo* purine synthesis is suppressed by the salvage of excess purine bases, a known allosteric regulation of purine synthesis^21^. While these data together do not support a role for guanine in the direct or indirect inhibition of 1-carbon metabolism, it is possible that the increase in guanine in cells treated with adenine also contributes to the toxicity of adenine. A graphical representation of simplified purine salvage and *de novo* synthesis is shown in Fig. 2d.

**Fig. 3:**
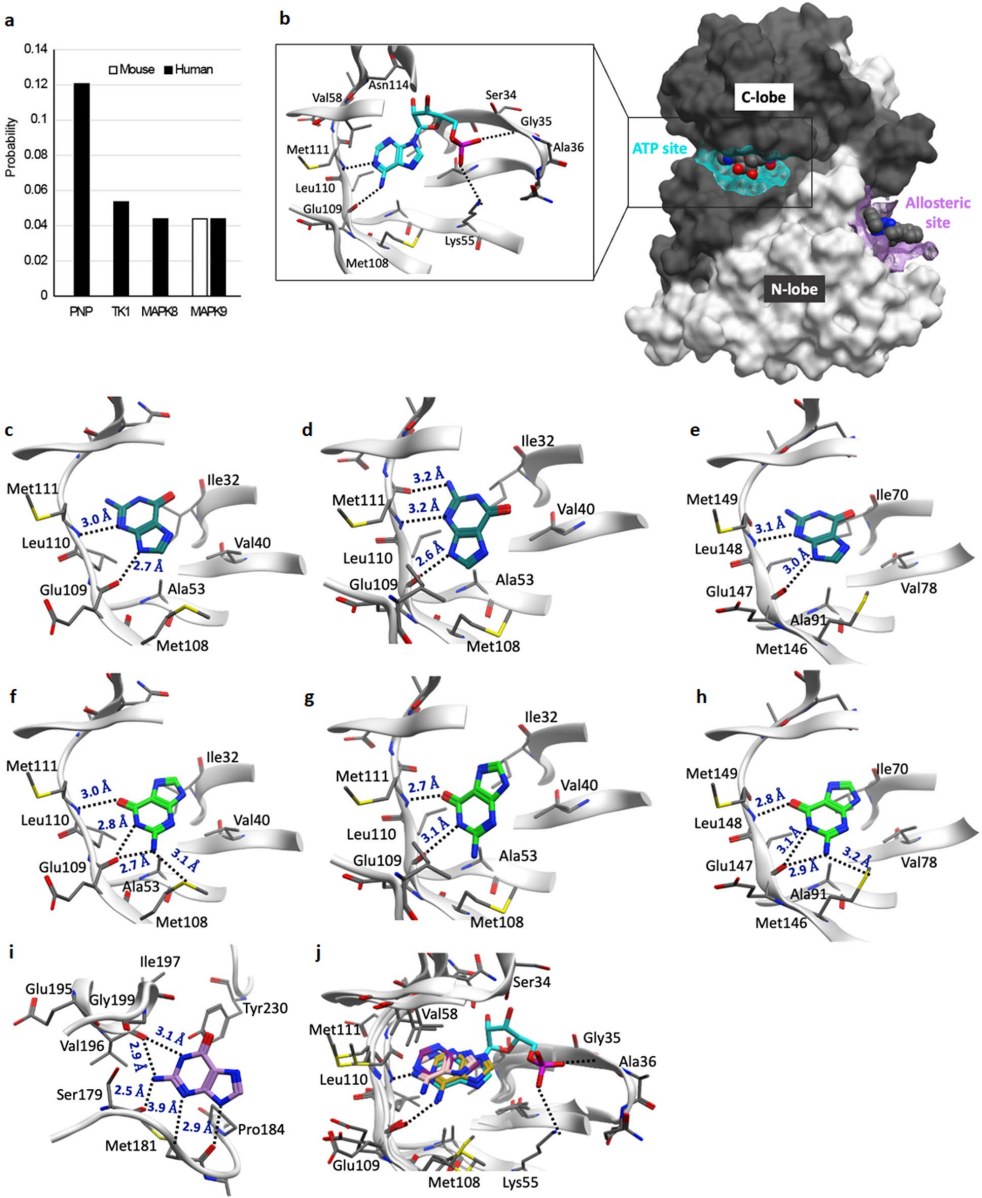
Prediction of guanine binding in the binding sites of JNKs.** a**, results of SwissTargetPrediction tool using human and mouse databases with guanine SMILES formula C1 = NC2 = C(N1)C(= O)NC(= N2)N. **b**, Space filling structure of JNK showing ATP binding site (cyan) using JNK2 X-ray structure (PDB ID: 7N8T^31^); and allosteric binding site (magenta) using JNK1 X-ray structure (PDB ID: 3O2M^33^). The detailed interatomic interactions of AMP (adenosine mono phosphate) in the ATP binding site of JNK2 (PDB ID: 7N8T^31^) is shown in the box. Detailed interatomic interactions of the top-ranked docked pose of guanine in the ATP-binding site of **c**, JNK1 (PDB ID: 2GMX^30^), **d**, JNK2 (PDB ID: 7N8T^31^) and **e**, JNK3 (PDB ID: 3G90^32^). Detailed interatomic interactions of the guanine in its flipped-orientation in the ATP-binding site of **f**, JNK1 (PDB ID: 2GMX^30^), **g**, JNK2 (PDB ID: 7N8T^31^) and **h**, JNK3 (PDB ID: 3G90^32^). **I**, Detailed interatomic interactions of the top-ranked docked pose of guanine in the allosteric binding site of JNK1 (PDB ID: 3O2M^33^). **j**, Superposition of the top-ranked docked poses of adenine in the ATP-binding site of JNK1 (violet, PDB ID: 2GMX^30^), JNK2 (gold, PDB ID: 7N8T^31^), JNK3 (pink, PDB ID: 3G90^32^) and AMP (cyan, PDB ID: 7N8T^31^). Dashed lines represent hydrogen bonds and values of the distance between heteroatoms in Å.

Next, to probe potential protein targets of guanine, we used SwissTargetPrediction^22^ with guanine, which revealed Mitogen-activated protein kinase 9 (MAPK9), also known as c-Jun N-terminal kinase (JNK2) as the top most likely target using mouse data (Probability 0.044), and both MAPK8 (JNK1, 0.044) and MAPK9 (JNK2, 0.044) as the top ranked third and fourth target using human data (Fig. 3a). While these probabilities seem low, the actual ranking of these potential targets is the most meaningful parameter^23^. For comparison, purine nucleoside phosphorylase (PNP) and thymidine kinase (TK1), enzymes respectively involved in purine and pyrimidine metabolism that are known to bind guanine or its analogues^24,25^were the top first (0.121) and second (0.053) targets using human data (Fig. 3a).

MAPK8 and MAPK9 are interesting potential targets because their siRNA-mediated knock-down and inhibition with the inhibitor SP600125 have been shown to lengthen the circadian rhythms in vitro^26^. JNKs are key kinases regulating development and cell growth, stress response, apoptosis and inflammation^27^. MAPK8 and MAPK9 are expressed in different tissues and implicated in various diseases, while MAPK10 is mainly expressed in brain and implicated in the pathogeneses of CNS disorders^28,29^.

The general structure of JNKs comprise a C-lobe and N-lobe domain (Fig. 3b). The ATP binding site is in the hinge region between the two lobes, involving more interaction with N-lobe amino acid residues. It is known JNK inhibitors either bind to the ATP binding pocket or to the allosteric site (Fig. 3b). To probe the potential for binding of guanine to JNKs, we performed docking studies using the ATP-binding pocket of the three kinases (from X-ray structures PDB ID: 2GMX^30^ for JNK1, 7N8T^31^ for JNK2, and 3G90^32^ for JNK3), and the allosteric site of JNK1 (PDB ID: 3O2M^33^). For all JNKs, the top-ranked docked pose of guanine was predicted to form hydrogen bonds in the cofactor binding pocket with residues similar to the adenine base of ATP (Fig. 3c-e). However, in docking, a flipped orientation of guanine was also predicted as favourable, with the potential to hydrogen bond with the gatekeeper residue (Met108 in JNK1,2 and Met146 in JNK3, Fig. 3f-h). Guanine was also predicted to fit with good affinity to the allosteric site of JNK1 with a ChemGauss4 value of −6.7 (Fig. 3i; Table 1). For comparison, adenine was docked into the ATP binding site of the JNK isoforms. The top-ranked docked poses of adenine in the three JNKs structures show binding poses similar to the adenine part of AMP (Fig. 3j), and possess with slightly lower docking scores than that of the top-ranked poses of guanine (Table 1). Together these data predict a relatively favourable interaction of guanine for all JNKs.

**Table 1:** ChemGauss4 Docking scores of X-ray ligand, guanine and adenine in ATP and non-ATP binding sites of JNK1, JNK2 and JNK3.

Isoform	PDB ID	Gatekeeper	X-ray Ligand	Guanine (top-ranked pose)	Guanine (flipped pose)	Adenine (top-ranked pose)
JNK1	2GMX^30^	Closed	−11.8	−7.9	−6.9	−6.9
JNK2	7N8T^31^	Closed	−12.1	−9.8	−7.2	−7.4
JNK3	3G90^32^	Closed	−11.9	−8.1	−5.9	−7.1
JNK1	3O2M^33^	N/A	−7.9	−6.7	-	-

**Fig. 4:**
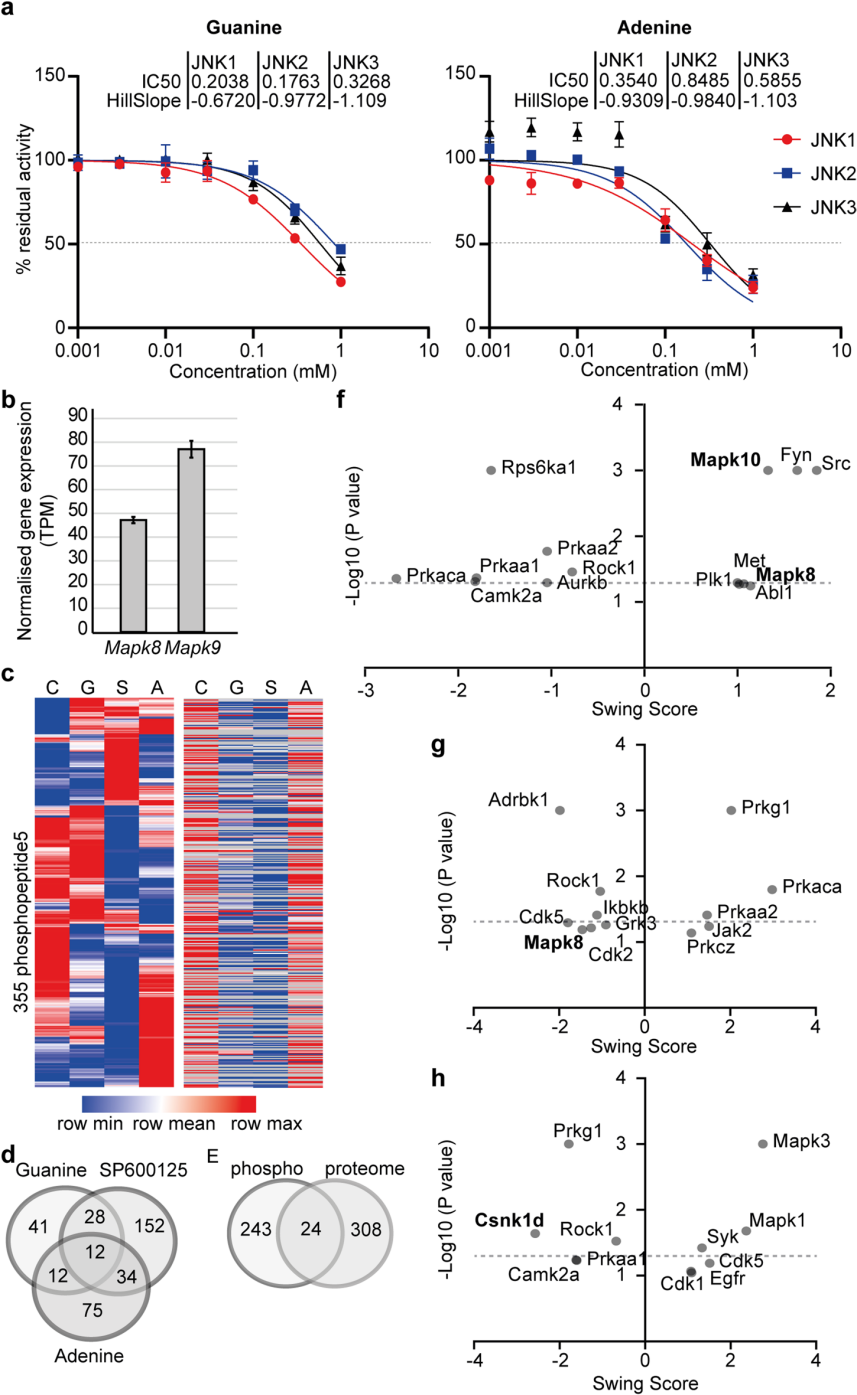
Guanine is an inhibitor of JNKs**. a**, Radiometric kinase assays using ATF2 as a substrate. Data show mean +/- SD of *n* = 3 independent experiments, with Hill slope and IC50 values extrapolated form the non-linear fit curve. **b**, mRNA expression of *Mapk8* and *Mapk9* in mouse embryonic fibroblasts, from previously published RNAseq data^16^. Data show mean +/- SD of *N* = 4 replicate wells. **c**, Phosphoproteome (left heatmap) and input proteome (right) of cells treated with vehicle (C), guanine (G), SP600125 (S) or adenine (A). Only the phosphopeptides significantly (*P* < 0.05) regulated in at least one treatment are shown. Data shows mean of *n* = 4 replicate dishes. **d**, Venn diagram with the number of significantly regulated phosphopeptides detected in each condition vs. control. **e**, Venn diagram showing the number of significantly (*P* < 0.05) regulated proteins and phosphoproteins. **f**, Graph showing KinSwingR results of kinases with a probability of observing such a swing score < 0.1 and their predicted swing scores, based on the phosphoproteome signatures of guanine-treated cells, adenine-treated cells (**g**), or SP600125-treated (**h**) cells. The gray dotted line indicate the significance threshold (probability < 0.05).

Next, we determined the inhibitory effects of guanine on JNK1-3 by radiometric in vitro kinase assays. As predicted, guanine inhibited all three JNKs, with an IC50 value of 0.2, 0.18 and 0.33 mM and Hill Slope of −0.67, −0.98 and − 1.1 for JNK1, 2 and 3, respectively (Fig. 4a). The lower IC_50_ and Hill slope for JNK2 are consistent with their more favourable predicted docking scores. In this assay, we also included adenine, as another purine base, and revealed some inhibition of all JNKs, but with a higher IC50 (Fig. 4a) than guanine, and in contrast weakest for JNK1.

Using our previous RNASeq dataset^16^we confirmed that the PER2::LUC cells we used express JNK1 and 2, the latter the most abundantly expressed homologue, but not JNK3 (Fig. 4b). We then quantified the phosphoproteome of PER2::LUC cells treated with 0.5 mM guanine or adenine for 48 h, to probe direct or indirect JNKs inhibition, respectively, or with 9 µM SP600125, a compound originally described as a specific JNK inhibitor but also a potent inhibitor of other kinases including Aurora and Casein kinases^34,35^. The complete (phospho)proteomics data have been deposited to the ProteomeXchange Consortium via the PRIDE^36^ partner repository with the dataset identifier PXD062305 and 10.6019/PXD062305.

We detected more than 5500 phosphopeptides (Table S1) but limited our downstream analysis to those 1433 detected in at least 70% of the samples (Table S2). Out of these, 355 phosphopeptides showed significant changes (*P* < 0.05) in at least one treatment (Fig. 4c), but with little overlap between the effects of guanine, adenine or SP600125 (Fig. 4d). Comparison between the phosphoproteome and input proteome data (Table S3) showed efficient enrichment for phosphopeptides, as many proteins with detected phosphopeptides were not detected in the proteome data, and did not reveal overall parallel changes (Fig. 4c), evidence that the changes in phosphopeptides were mostly due to changes in phosphorylation rather than protein abundance. Indeed, performing the same differential analysis by PhosPIR but with the proteome revealed 332 proteins significantly regulated in at least one treatment (*P* < 0.05, Table S4), but only 24 of these proteins had significantly regulated phosphopeptides in the phosphoproteome, with the other 243 proteins with significant phosphopeptides not represented among significant proteins (Fig. 4e).

To predict which kinase(s) might be regulated by guanine, adenine or SP600125, we then performed analysis of our phosphoproteome with KinSwingR^37^. This analysis revealed that MAPK10 had a significant (*P* < 0.05) swing score (change in activity) of 1.33 and MAPK8 a swing score of 1.14 with a P value of 0.057 in guanine-treated cells (Fig. 4f; Table S5). MAPK9 had a poor swing score and a P value > 0.1. Surprisingly, however, the swing scores for MAPK8 and 10 were positive, indicating an increase in their activity. This is consistent with higher levels of the canonical target JUN S73 phosphorylation in guanine-treated cells compared to control (Table S6). Although this may appear contradictory with their inhibition, it has been shown that while knock-out of MAPK8 decreases JUN phosphorylation and stability, MAPK9 deficiency or inhibition increases JUN expression, phosphorylation and stability due to a compensatory increase in MAPK8 activity^38,39^. Together with the higher expression of MAPK9 in our cells, these observations suggest that MAPK9 was the main homologue inhibited by guanine in our cells (Fig. 4b), in line with higher guanine docking affinity (Table 1) and lower EC50 (Fig. 4a).

In adenine-treated cells, a negative swing score of −1.46 for MAPK8 with a P value of 0.065 was obtained, but MAPK9 and MAPK10 had poor swing score and P value above 0.1 (Fig. 4g; Table S6). In cells treated with SP600125, while the swing scores for MAPK8-10 were poor and their P values was above 0.1, a negative swing score of −2.57 for Casein kinase 1 delta (CSNK1D) with a P value of 0.02 was observed (Fig. 4h; Table S7), which is consistent with a report showing that SP600125 is a more potent inhibitor of CSNK1D than of JNKs^34^.

**Fig. 5:**
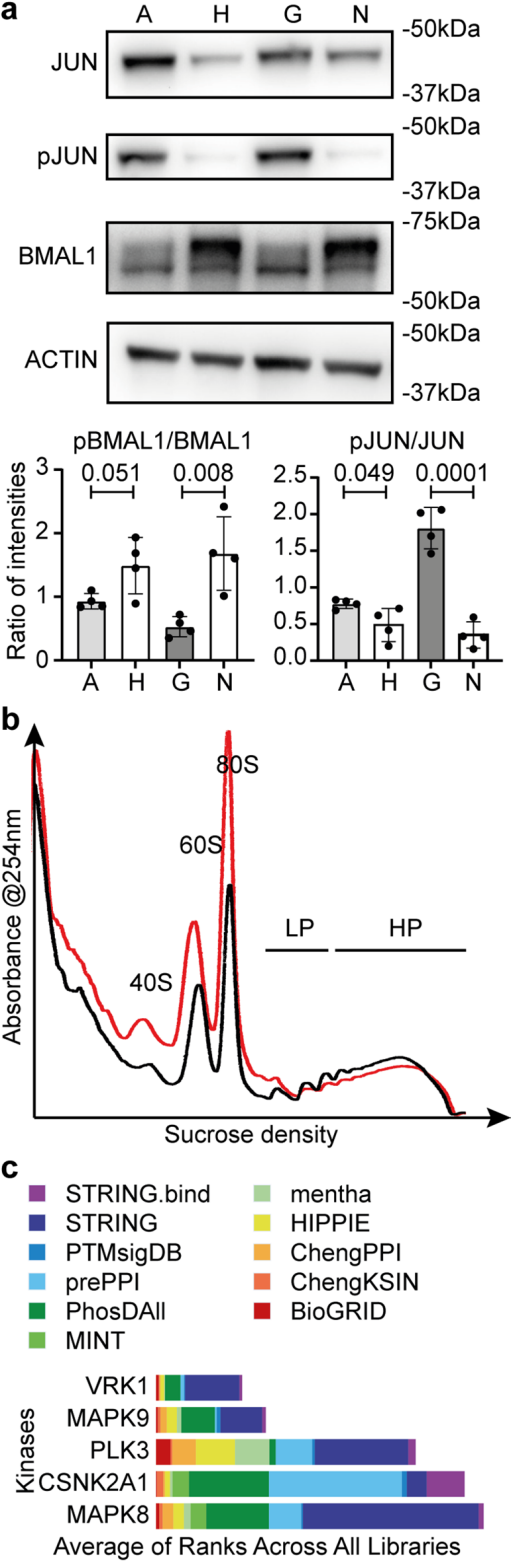
Consequences of JNKs inhibition by guanine and potential relevance in a cellular model of Lesch-Nyhan.** a**, Representative immunoblotting (experiment performed at least 3 times independently) results with PER2::LUC MEFs showing adenine (A) and guanine (G) cause higher abundance of JUN and pJUN but less intense upshifts in BMAL1 compared to their respective controls HCl (H) and NaOH (N). Quantification of phosphorylated/native ratio of band intensities is shown underneath, analysed by t-tests with P values indicated above the bars, showing mean +/- SD of *N* = 4 replicate cell extracts. **b**, Representative (experiment performed at least 3 times independently) profiling traces showing a pronounced decrease in light and heavy polysome (LP, HP) associated with an increase in free 60 and 80 S ribosome particles in with PER2::LUC MEFs treated for 24 h with 0.5 mM guanine (red), compared to cells treated with vehicle (black). For visualisation purpose, traces were aligned to the trough point between 40 S and 60 S, but aligning the trace to the lowest point of the heavy polysomes would lead to the same conclusion, i.e. an excess in free ribosome particles not involved in translation. **c**, Output of the Expression2Kinases Appyter using the data from Dammer et al. (2015), using dopaminergic rat PC6-3 lines carrying mutations in HPRT, showing the top five kinases predicted to explain changes in the proteome of HPRT mutant cells. The colours and size of compound bars respectively represent the different libraries used (names in legend) and scores given to that kinase by these libraries.

To confirm these observations, an increase in JUN and pS73 JUN by immunoblotting was seen in cells treated for 48 h with guanine or adenine (Fig. 5a), suggesting both treatments affect MAPK8-10 activity. Since MAPK8 and MAPK9 are involved in the regulation of circadian rhythms via the phosphorylation of the core clock protein BMAL1^26^, we sought to confirm whether BMAL1 phosphorylation was inhibited in guanine- or adenine-treated cells. Indeed, in cells treated with guanine or adenine, the intensity of an upper band, likely corresponding to phosphorylated BMAL1, markedly decreased in intensity (Fig. 5a, Figure S1) compared to their respective controls. Together these data demonstrate that guanine, and maybe adenine directly or indirectly, is an inhibitor of MAPK8-10.

Lastly, since MAPK9 promotes translation^40^we decided to assess the translation efficiency in guanine-treated cells to provide functional consequences of MAPK9 inhibition. Polysome profiling revealed a deficiency in translation, with an increase in free ribosomal 80 S subunits, associated with markedly lower polysome levels (Fig. 5b).

## Discussion

We have shown here that guanine is capable of inhibiting the function of JNKs, which may be of clinical significance for patients with genetic or life-style-related deficiencies in purine metabolism. To probe this possibility further, we mined a published proteomics dataset obtained with a model of Lesch-Nyhan disease based on dopaminergic rat PC6-3 lines carrying different mutations in HPRT^41^. Proteins affected by HPRT mutations^41^ were used as input for the Expression2Kinases Appyter that predicts upstream kinases likely responsible for observed changes in gene expression^42^. Interestingly, MAPK8 and MAPK9 were respectively fifth and second topmost likely kinases (Fig. 5c).

In cells, ATP is typically in the mM range, meaning that potent ATP-competitive kinase inhibitors typically have a K_i_ in the low micromolar range^43^. In vivo conditions in which JNKs inhibition by guanine could occur will thus depend on the levels of both guanine and ATP. Interestingly, HPRT knock-out neural progenitor cells have lower ATP levels^44^suggesting HPRT mutations may present perfect conditions for JNKs inhibition. The IC50 of guanine for JNK2 is 0.18 mM, which is high compared to pharmacological inhibitors operating at the nM range. This may be however pathologically important considering that 0.41 mM or above is the threshold uric acid plasma concentration for gout diagnosis, suggesting such high concentrations of purine bases may occur in human patients.

Our phosphoproteome data do no conclusively demonstrate which JNK isoform is inhibited, because their few substrate phospho-sites in the database used by PhosPIR largely overlap. In our transcriptome data, however, there was evidence that MAPK9 is the dominant homologue expressed in these cells, followed by MAPK8, with MAPK10 being undetectable. In our proteome data (Table S3), Maxquant was unable to confidently differentiate between MAPK8, 9 and 10, even though one of the two peptides detected, MLVIDPDKR, is specific to MAPK9, with only one amino acid difference with MAPK10. While comparative studies have concluded that MaxQuant has better overall performance than other commercial protein quantification tools^45,46^we decided to re-analyse our MS data with Thermo Fisher Scientific’s Proteome Discoverer (PD), which has been shown to provide better coverage of low abundance proteins^46^. PD was able to confirm that MAPK9 was the only JNK to be detected with high confidence in all samples (Table S8 and S9). This further supports the conclusion that, in our PER2::LUC cells, MAPK9 was the main homologue inhibited by guanine. Moreover, our data do not refute the possibility the guanine (and adenine) may also inhibit other kinases.

Our docking predictions (Fig. 3) and kinase assays (Fig. 4a) were based on human JNK sequences and recombinant proteins, while our other results were produced in mouse cells. Since human JNK1 is 100% identical to mouse JNK1, and since there is only one amino acid difference between mouse and human JNK2, away from the ATP binding domain, docking predictions and kinase assay data are also applicable to mouse JNKs.

The catabolism of guanine starts with guanine deaminase (GDA), leading to xanthine, further catabolised to urate by xanthine dehydrogenase (XDH). The development of gout and kidney stones in Lesch-Nyhan patients indicates that salvage of guanine to GMP by HPRT is the main metabolic route for guanine in normal conditions. HPRT deficiency causes elevated levels of it substrates guanine, hypoxanthine and PRPP, especially in the central nervous system^47,48^. Although GDA may mitigate the increase in guanine in the brain by degrading it to xanthine^49^it is expressed in neurons but not glia^50^raising the possibility that local, cell-specific accumulation of guanine may occur in Lesch-Nyhan patients, leading to JNKs inhibition as a potential contributor to the symptoms of the disease. This may be in a way similar to what happens in PER2::LUC MEFs treated with guanine. Indeed, from our previously published RNASeq^16^ data and the proteome data presented here, of the purine metabolism enzymes shown in Fig. 2d, GDA and XDH were not detected, indicating exogenous guanine could not be degraded. It would be interesting to express GDA in these cells to determine whether this provides protection against guanine, since the endogenous expression of HPRT in these cells was not sufficient to protect against exogenous guanine.

Guanine, like adenine, caused the lengthening of the circadian period in vitro but our results indicate the mechanisms underlying these effects are different. While adenine acts as a feedback inhibitor of 1-carbon metabolism and methylations, guanine instead inhibits JNKs, leading to the changes in BMAL1 electrophoretic mobility observed in Fig. 5a, likely attributable to phosphorylation^26^. Within the molecular clockwork, phosphorylation of BMAL1 and its partner CLOCK is circadian-time dependent and inhibits its activity at E-boxes cis-elements in the promoter of target genes^26,51,52^. A delay in this phosphorylation-dependent inactivation would lead to a lengthened circadian period, here observed with guanine or previously published when JNKs are inhibited or knocked out/down^26^.

The observed increase in JUN phosphorylation and the decrease in BMAL1 electromobility upshift in cells treated with adenine or guanine may appear contradictory with an inhibition of JNKs. Unlike JUN that can be phosphorylated by both JNK1 and JNK2, with the compensatory increase in JNK1 when JNK2 is inhibited responsible for the increase in pJUN observed^38,39^it is possible that BMAL1 may be phosphorylated specifically by JNK2, and therefore not affected by the compensatory increase in JNK1. This would ultimately lead to a decrease in BMAL1 phosphorylation due to JNK2 inhibition.

In the phosphoproteome analysis from cells treated with guanine (Fig. 4f), of note was the negative swing score of both PRKAA1, PRKAA2, subunits of 5’ AMP-activated protein kinase (AMPK). These subunits may have been directly inhibited by guanine, or may have been inhibited indirectly by potential changes in the abundance of adenosine nucleotides (ATP, ADP, AMP), known regulators of AMPK, potentially caused by treatment with guanine or adenine^53^. In cells treated with adenine, in contrast PRKAA2 had a positive swing score, suggesting AMPK may have been activated instead (Fig. 4f). It is possible that excess of guanine nucleotides from salvage in guanine-treated cells may have stimulated the *de novo* branch of the pathway to ATP, while treatment with adenine instead may have inhibited ATP production. Of note, AICAR, an activator of AMPK^54^was lower in cells treated with guanine or adenine (Fig. 2c). It is known that AMPK and JNK pathways interact under metabolic stress^55^which may have further contributed to the changes in the phosphoproteome observed here. Of note, AMPK itself is a regulator of circadian rhythms^56^and changes in AMPK activity may also have contributed to the long circadian period observed in cells treated with adenine or guanine.

In addition to the potential contributions of changes in nucleotide pools and energy metabolism, the effects of guanine and adenine we report here may have been in part mediated by purinergic receptors. Although nucleoside and nucleotides are the endogenous ligands of these receptors, some of these receptors have been reported to bind adenine and guanine^57,58^.

In conclusion, we have shown here that inhibition of JNK contributes to the toxicity of purine bases, and further explains why the biosynthesis of purine bases is under strict control.

## Experimental procedures

### Cell cultures

All procedures were carried out under the UK Animals (Scientific Procedures) Act (1986) and approved by the University of Manchester Review Ethics Panel. PER2::LUC MEFs were prepare from heterozygous PER2::LUC embryos^17^ at day 11.5–14.5 from a dam euthanised by cervical dislocation followed by cessation of circulation. The uterus was excised and placed in sterile ice-cold PBS in a Petri dish, and each single embryo was freed form maternal tissues and transferred to its own Petri dish with sterile ice-cold PBS. Internal organs and head were removed from the embryos (kept to confirm genotype) and embryos were washed by two transfer into new Petri dishes containing sterile ice-cold PBS. Each embryo was then transferred to a Petri dish containing 1x Trypsin, finely minced with scissors and scalpel and incubated 15 min in a cell culture incubator (37 °C, 5% CO_2_). Minced embryos were then centrifuged at 1000 xg for 5 min, resuspended in 10 ml DMEM/F12 medium (Invitrogen) containing antimycotic/antibiotic (Sigma) and 10% heat-inactivated serum (Gibco) and incubated in a 10 cm Petri dish for a few days, during which cells will migrate out and onto the plate. From that point, PER2::LUC MEFs were passaged every 3–4 days and will spontaneously immortalise after several weeks, at which point lines (each line comes from a single embryo) showing stable luminescence rhythms are selected for downstream experiments and/or frozen in liquid nitrogen for storage.

PER2::LUC MEFs were cultivated and monitored for real-time luminescence as previously described^20^. Briefly, cells were seeded into 35 mm dishes (Corning) and allow to grow for 3–4 days to confluence in DMEM/F12 medium (Invitrogen) containing antimycotic/antibiotic (Sigma) and 10% heat-inactivated serum (Gibco). Cells were shocked with 400 nM dexamethasone (Sigma) for 2 h, followed by a medium change including 1 mM beetle luciferin (Promega) and either of the following treatments: adenine, guanine, xanthine and hypoxanthine (Sigma), keeping the concentration of the respective vehicle equal in all dishes (1.6 µM HCl for adenine, 1.6 µM NaOH for guanine, xanthine and hypoxanthine). 35 mm dishes were then sealed with parafilm and transferred to a luminometer (Lumicycle32, Actimetrics) placed in an dry incubator at 35 °C. Photons were counted in bins of 2 min at a frequency of 10 min. Period and amplitude were estimated by BioDare2^59^.

### Metabolite quantification by LC-MS/MS

PER2::LUC MEFS^17^ were cultivated and metabolites were extracted as previously described^20^. To repeat, cells cultivated in 10 cm Petri dishes (Corning) for 3–4 days until confluence were treated with guanine, adenine, HCl or NaOH and returned to the incubator for 24 h at 37 °C, 5% CO2. Cells were washed twice with 10 ml 5% mannitol (Sigma), the mannitol was carefully and completely removed before 0.9 ml 100% methanol was added onto the cells, firmly rocking the dish so that the methanol covers the cell monolayer. Dishes were tipped, and 0.6 ml water containing 125 ng/ml BIS-TRIS (Sigma) was added directly into the pool of methanol forming in the corner of the dish before rocking the dish again to cover the cell monolayer. The water/methanol mix was collected from the corner of the tipped dish and transferred to a 1.5 ml microtube. Tubes were kept at room temperature until all dishes were processed, randomly. Samples were centrifuged at 20,000 × g, 4 °C for 30 min, the supernatant transferred to a new tube, centrifuged again at 20,000 × g, 4 °C for 10 min, and the final supernatant transferred to a new 1.5 ml microtube.

Prior to analysis, 200 µl of sample was dried in a centrifugal vacuum concentrator and resuspended in 100 µl acetonitrile and water in a ratio of 5:1. The sample was centrifuged at 20,000 × g for 3 min and the top 80 µl was transferred to a glass autosampler vial with 300 µl insert and capped.

Liquid chromatography-mass spectrometry analysis was performed using a Thermo-Fisher Ultimate 3000 HPLC system consisting of an HPG-3400RS high-pressure gradient pump, TCC 3000 SD column compartment, and WPS 3000 Autosampler, coupled to a SCIEX 6600 TripleTOF Q-TOF mass spectrometer with TurboV ion source. The system was controlled by SCIEX Analyst 1.7.1, DCMS Link, and Chromeleon Xpress software.

A sample volume of 5 µL was injected by pulled loop onto a 5 µL sample loop with 150 µl post-injection needle wash with 9:1 acetonitrile and water. Injection cycle time was 1 min per sample. Separations were performed using an Agilent Poroshell 120 HILIC-Z PEEK-lined column with dimensions of 150 mm length, 2.1 mm diameter, and 2.7 μm particle size equipped with a guard column of the same phase. Mobile phase A was water with 10 mM ammonium formate and 0.1% formic acid, mobile phase B was 9:1 acetonitrile and water with 10 mM ammonium formate and 0.1% formic acid. Separation was performed by gradient chromatography at a flow rate of 0.25 ml/min, starting at 98% B for 3 min, ramping to 5% B over 20 min, hold at 5% B for 1 min, then back to 98% B. Re-equilibration time was 5 min. Total run time including 1 min injection cycle was 30 min.

The mass spectrometer was run in positive mode under the following source conditions: curtain gas pressure, 50 psi; ionspray voltage, 5500 V; temperature, 400 °C; ESI nebulizer gas pressure, 50 psi; heater gas pressure, 70 psi; declustering potential, 80 V.

Data were acquired in a data-independent manner using SWATH in the range of 50–1000 m/z, split across 78 variable-size windows (79 experiments including TOF survey scan), each with an accumulation time of 20 ms. Total cycle time was 1.66 s. Collision energy of each SWATH window was determined using the formula CE (V) = 0.084 × m/z + 12 up to a maximum of 55 V.

Acquired data were processed in MultiQuant 3.0.2. Peaks from MS1 and MS2 data were picked and matched against a metabolite library of 235 standards, based on retention time and mass error of ± 0.025 Da. Data exported from MultiQuant 3.0.2 was further sorted, filtered, and scored using a custom VBA macro in Excel, based on presence, peak area, and coelution of precursor and fragment ions.

### Phospho-proteomic analysis and mass spectrometry in BioMS

Samples (frozen cell pellets from 4 independent replicate 15 cm Petri dishes per treatment) were lysed in 5% SDS, followed by reduction, alkylation and precipitation with acetone. Samples were then resuspended in Rapigest (Waters) and digested with trypsin overnight. For phospho-peptide enrichment, 95% of each sample volume was desalted (TELOS neo SPE fixed 96-well plates, Cole-Parmer) according to standard protocols, and eluted in phophopeptide enrichment binding solution for processing. The remaining 5% of the digested sample was taken for proteomic analysis. Phospho-peptide enrichment was performed using magnetic microspheres (Ti-IMAC, ReSyn Bioscience) and a KingFisher Flex (Thermo Scientific) according to facility protocols^60^. Phospho-peptides were desalted prior to analysis (TELOS neo SPE fixed 96-well plates, Cole-Parmer).

For mass spectrometry peptides were resuspended in 3% (v/v) ACN/1% (v/v) formic acid and analysed by liquid chromatography-tandem mass spectrometry (LC-MS/MS) using a Thermo Rapid Separation Liquid Chromatography system (RSLC, Thermo Fisher Scientific) coupled to an Exploris 480 (Thermo Fisher Scientific) mass spectrometer.

The RSLC was configured with buffer A as 0.1% formic acid in water and buffer B as 0.1% formic acid in acetonitrile. An injection volume of 2 ul was loaded into the end of a 5 ul loop and reverse flushed on to the analytical column (Waters nanoEase M/Z Peptide CSH C18 Column, 130 Å, 1.7 μm, 75 μm X 250 mm) kept at 35 °C at a flow rate of 300 nl/min for 8 min with an initial pulse of 500 nl/min for 0.3 min to rapidly re-pressurise the column. The injection valve was set to load before a separation consisting of a multistage gradient of 2% B to 6% B over 3 min, 6% B to 18% B over 67 min, 18% B to 29% B over 11 min and 29% B to 65% B over 1 min before washing for 6 min at 65% B and dropping down to 2% B in 1 min. The complete method time was 105 min.

The analytical column was connected to a Thermo Exploris 480 mass spectrometry system via a Thermo nanospray Flex Ion source via a 20 μm ID fused silica capillary. The capillary was connected to a stainless steel emitter with an outer diameter of 150 μm and an inner diameter of 30 μm (Thermo Scientific, ES542) via a butt-to-butt connection in a steel union using a custom made gold frit (Agar Scientific AGG2440A) to provide the electrical connection. The nanospray voltage was set at 1900 V and the ion transfer tube temperature set to 275 °C.

Data was acquired in a data dependent manner using a fixed cycle time of 2 s, an expected peak width of 15 s and a default charge state of 2. Full MS data was acquired in positive mode over a scan range of 300 to 1750 Th, with a resolution of 120,000, a normalised AGC target of 300% and a max fill time of 25 mS for a single microscan. Fragmentation data was obtained from signals with a charge state of + 2 or + 3 and an intensity over 5,000 and they were dynamically excluded from further analysis for a period of 15 s after a single acquisition within a 10 ppm window. Fragmentation spectra were acquired with a resolution of 15,000 with a normalised collision energy of 30%, a normalised AGC target of 300%, first mass of 110 Th and a max fill time of 25 mS for a single microscan. All data was collected in profile mode. The complete (phospho)proteomics raw data have been deposited to the ProteomeXchange Consortium via the PRIDE^36^ partner repository with the dataset identifier PXD062305 and 10.6019/PXD062305.

Raw files were analysed by MaxQuant v2.6.7.0, using parameters listed in the Method S1 using the UP000000589_10090 fasta reference proteome file available form uniprot.org. The Phospho(STY)Sites.txt output file (Table S1) was further analysed using PhosPIR^62^limited to the phosphopeptides detected in at least 70% of the samples, normalising the data and imputing the remaining missing values, and using significance cutoff value of *P* < 0.05. For statistical analysis with PhosPIR including KinSwingR, the 3 pairwise comparisons guanine vs. control, adenine vs. control and SP600125 vs. control were setup. Protein identification and quantification by Proteome Discoverer version 3.1.0.638 (Table S8) was performed using the settings listed in Table S9, which also include workflow messages.

### Polysome profiling

Sucrose gradients were made by layering 2 ml of successive 50%, 40%, 30%, 20% and 10% sucrose solutions made in 100 µM HEPES-KOH, 25µM MgCl2 (Sigma), 22.5 mM KCl (Sigma) and 0.625 U/µl RNasin plus RNase inhibitor (Promega) in 12 ml ultracentrifuge tubes (SETON Scientific), freezing each layer in liquid nitrogen before adding the next layer. The tubes were sealed and kept at 80 °C until use. The day before the experiments, tubes were thawed upright at 4 °C overnight.

PER2::LUC MEFs were plated in 10 cm Petri dishes and left to reach confluency. Once confluent, the plates were incubated (37 °C, 5% CO2) for 24 h in DMEM/F-12 containing 0.5 mM guanine or 1.6 µM NaOH. Medium was then removed and cells were incubated 5 min in ice-cold PBS (Fisher, BP399) containing 100 µg/ml cycloheximide (Sigma). Cells were washed once more with ice-cold PBS containing 100 µg/ml cycloheximide, then scraped in 1 ml ice-cold PBS containing 100 µg/ml cycloheximide using a rubber policeman and transferred to pre-cooled 2 ml tubes. Cells were centrifuged for 5 min at 500 xg at 4 ^o^C, the supernatant was removed and the cells were lysed in polysome lysis buffer containing 100 µM HEPES-KOH, 25 µM MgCl2, 22.5 mM KCl, 250 nM DTT (Sigma), 40 U/µl RNasin plus RNase inhibitor, 100 µg/ml cycloheximide, 0.005% NP40 substitute (ITWreagents, A1694) and EDTA-free protease inhibitor (cOmplete, Roche) for 10 min on ice. The lysates were centrifuged for 10 min at 1300 xg at 4 ^o^C. The absorbance at 260 nm (NanoDrop 1000, Thermo Scientific) of each lysate was measured and the concentrations of all lysate were equalised. The samples were centrifuged again for 5 min at 1300 xg at 4 ^o^C, aliquoted and kept at −20 ^o^C until use. Cells lysates (200 µL) were layered unto the sucrose gradient and ultracentrifuged at 36,000 RPM for 2 h at 4 ^o^C (lowest acceleration, no brake, SW41 Ti rotor, Beckman Optima XE90). Polysome profiles were generated by continuous *A*_254_ recording using a UA-6 UV/Vis detector and analogue chart recorder (Teledyne ISCO).

### Kinase assays

Kinase assays were performed independently by Reaction Biology (RBE, Freiburg, Germany) as a service, with their own products and according to their standard protocol, as follows.

All JNKs (human untagged JNK1 (amino acids 1-384, product code 0458-0000-1 lot 5), untagged JNK2 (amino acids 1-424, product code 0459-0000-1 lot 3) and GST-tagged JNK3 (amino acids 1-426, product code 0900-0000-1 lot 4)) were expressed in *E.coli*. The purity of the JNKs was examined by SDS-PAGE/Coomassie staining. Adenine (Sigma), guanine (Sigma) and SP600125 (Sigma) were dissolved and diluted in 100% DMSO to 100x highest assay concentration (1 mM, 1 mM, 2 µM, respectively). Prior to testing, the 100% DMSO stock solutions were subjected to a serial, semi-logarithmic dilution using 100% DMSO as a solvent.

RBE used their own radiometric protein kinase assay^33^PanQinase™ Activity Assay) for measuring the kinase activity of the three JNKs. All kinase assays were performed in 96-well ScintiPlates™ from PerkinElmer (Boston, MA, USA) in a 50 µl reaction volume. The reaction cocktail was pipetted in four steps in the following order: 1, 25 µl of assay buffer (standard buffer/[g-33P]-ATP); 2, 10 µl of ATP solution (in H2O); 3, 5 µl of test compound (in 10% DMSO); 4,10 µl of enzyme/substrate mixture. The assay contained 70 mM HEPES-NaOH, pH 7.5, 3 mM MgCl2, 3 mM MnCl2, 3 µM Na-orthovanadate, 1.2 mM DTT, 50 µg/ml PEG20000, ATP (variable concentrations, corresponding to the apparent ATP-Km of the respective kinase, i.e. 0.3 µM for JNK1, 1.0 µM for JNK2, 0.3 µM for JNK3), [g −33P]-ATP (~ 3 × 10^5^ cpm per well), protein kinase (2.3 nM, 2.0 nM, 2.1 nM for JNK1-3 respectively), and substrate (ATF2, 0.25 µg, 1.0 µg, 2.0 µg for JNK1-3 respectively). The reaction cocktails were incubated at 30 °C for 60 min. The reaction was stopped with 50 µl of 2% (v/v) H_3_PO_4_, plates were aspirated and washed two times with 200 µl 0.9% (w/v) NaCl. Incorporation of^33^Pi was determined with a microplate scintillation counter (Microbeta, Wallac).

The median value of the counts in wells without enzyme or test compounds (low control) was subtracted from the median value of the counts in wells with enzyme but without test compounds (high control) to obtain a measure of 100% activity, and from all the other values obtained with added compounds withing the same plate. The residual activity (in %) for each well of a given plate was calculated by using the following formula: Res. Activity (%) = 100 X [(cpm of compound – low control)/(high control – low control)]. The residual activities for each concentration and the compound IC50 values were calculated using *Quattro Workflow V3.1.1* (Quattro Research GmbH, Munich, Germany; www.quattro-research.com). The fitting model for the IC50 determinations was “Sigmoidal response (variable slope)” with parameters “top” fixed at 100% and “bottom” at 0%. The fitting method used was a least-squares fit.

As a parameter for assay quality, the Z´-factor^63^ for the low and high controls of each assay plate was used. RBE´s criterion for repetition of an assay plate is a Z´-factor below 0.4^64^. As an additional quality control, a control inhibitor (Staurosporine) was tested in parallel. The inhibitor IC50 values were in the expected range for each kinase.

### Immunoblotting

Proteins were visualised by Western blot as previously described^16^ with some modifications. Confluent PER2::LUC MEFs cultivated in 24-well plates were treated with 0.5 mM guanine, 0.5 mM adenine or respective vehicles (for 48 h in the incubator at 37 °C, 5% CO2. Cells were washed once with 1 ml PBS then lysed in the plate with 0.1 ml/well 2X Laemmli buffer (Bio-Rad) supplemented with 20 mM DTT. Cells were scraped out with a pipette tip and transferred to a 1.5 ml microtube, boiled for 10 min at 95 °C, vortexed at full speed for 8 s, and spinned-down before being split into single-use aliquots kept at −20 °C. On the day of the immunoblotting, aliquots were boiled again for 10 min at 95 °C, vortexed at full speed for 5–10 s, and spinned-down.

Samples (10 µl/well) were loaded into a pre-cast mini-PROTEAN gel (Bio-Rad), run and transferred in a min Trans-blot cell according to manufacturer’s instructions and consumables (Bio-Rad). Membranes were probed with primary antibodies against cJUN (60A8, Cell Signalling #9165 lot 13, 1247 citations, 1:1000), S73p-cJUN (D47G9, Cell Signalling #3270 lot 5, 449 citations, 1:1000), BMAL1 (D2L7G, Cell Signalling #14020 lot 4, 89 citations, 1:1000), Actin (Sigma A5441, > 10,000 citations, 1:5000) and secondary (anti-rabbit, Amersham NA934, 1:10,000; anti-mouse, Amersham NA931, 1:50,000) antibodies overnight at 4 °C–1 h at room temperature, respectively, and followed by three washes of 10 min at room temperature with Tris Buffered Saline 0.1% Tween20. Proteins were detected using chemiluminescent substrate (Amersham ECL-Prime), pictures of membranes were acquired with a G: Box (Syngene).

Integrated densities of bands were measured by ImageJ version 1.54p, and the phosphorylated/native ratios, top/bottom within the same membrane for BMAL1 or pJUN/JUN from their respective membranes, were calculated for each replicate samples.

### Molecular docking

The structures of JNK1 (PDB ID: 2GMX^30^3O2M^33^), JNK2 (PDB ID: 7N8T^31^) and JNK3 (PDB ID: 3G90^32^) were prepared using MOE^65^ and used for computational docking. Docking was performed using the OpenEye^66^ software suite. The OMEGA classic tool^67^ was used to create a 3D structure of the compounds using maximum conformations of 500 for each compound. The binding sites were prepared for docking using the make receptor module. The FRED^68,69^ module was used to dock the compounds using the ChemGauss4 scoring function. The best 50 poses for each compound were visualized using Vida 4.4.0^66^ and MOE 2024.06^65^. The docking process was validated by redocking of the cocrystallized ligand in the binding site for each JNK, where the ligand docked poses were found to reproduce the X-ray orientation.

## Electronic supplementary material

Below is the link to the electronic supplementary material.

Supplementary Material 1

Supplementary Material 2

Supplementary Material 3

Supplementary Material 4

## Data Availability

The complete (phospho)proteomics raw data have been deposited to the ProteomeXchange Consortium via the PRIDE(36) partner repository with the dataset identifier PXD062305 and 10.6019/PXD062305. All non-omics data related to this manuscript can be requested form the lead corresponding author at jean-michel.fustin@manchester.ac.uk.
