# Supplementary material for: Guanine is an inhibitor of c-jun terminal kinases
Source: Sci Rep. 2025 Aug 11;15:29374. doi: 10.1038/s41598-025-11617-3 (PMC12340087; doi:10.1038/s41598-025-11617-3)
Supplement: Supplementary file 2 — Supplementary Material 2 [file 41598_2025_11617_MOESM2_ESM.docx]

**Guanine is an inhibitor of c-Jun terminal kinases**

Jessica Treeby^1*^, Sherihan El-Sayed^2,3^, Samuel Morgan^1^, Sophie Maddock^1^, George Taylor^4^, Stacey Warwood^4^, Julian Selley^4^, David Knight^4^, Benjamin Saer^1^, Richard A. Bryce^2*^, Jean-Michel Fustin^1*^

^1^The University of Manchester; Faculty of Biology, Medicine and Health; Centre for Biological Timing

^2^ Division of Pharmacy and Optometry, School of Health Sciences, Manchester Academic Health Sciences Centre, University of Manchester, Oxford Road, M13 9PT, UK.

^3^ Department of Medicinal Chemistry, Faculty of Pharmacy, Zagazig University, Zagazig, 44519, Egypt.

^4^ The University of Manchester, FBMH Platform Sciences, Enabling Technologies & Infrastructure, BioMS

^*^Correspondence: Jessica.Treeby@postgrad.manchester.ac.uk, Jean-Michel.Fustin@Manchester.ac.uk, Richard.Bryce@manchester.ac.uk.

List of material included:

Supporting data 1

Table S1: Phospho (STY)Sites output from MaxQuant.

Table S2: PhosPIR output, using Phospho (STY)Sites as input

Table S3: ProteinGroups output from MaxQuant

Table S4: PhosPIR output, using ProteinGroups values as input

Table S5: Guanine vs Control output of KinSwingR

Table S6: Adenine vs Control output of KinSwingR

Table S7: SP600125 vs Control output of KinSwingR

Table S8: raw output from Proteome Discoverer

Table S9: PS methodology and settings applied to Proteome Discoverer

Figure S1: Uncropped membranes for Figure 5a

Method S1: xml settings file used with MaxQuant
