# Supplementary figures and images for: Guanine is an inhibitor of c-jun terminal kinases
Source: Sci Rep. 2025 Aug 11;15:29374. doi: 10.1038/s41598-025-11617-3 (PMC12340087; doi:10.1038/s41598-025-11617-3)

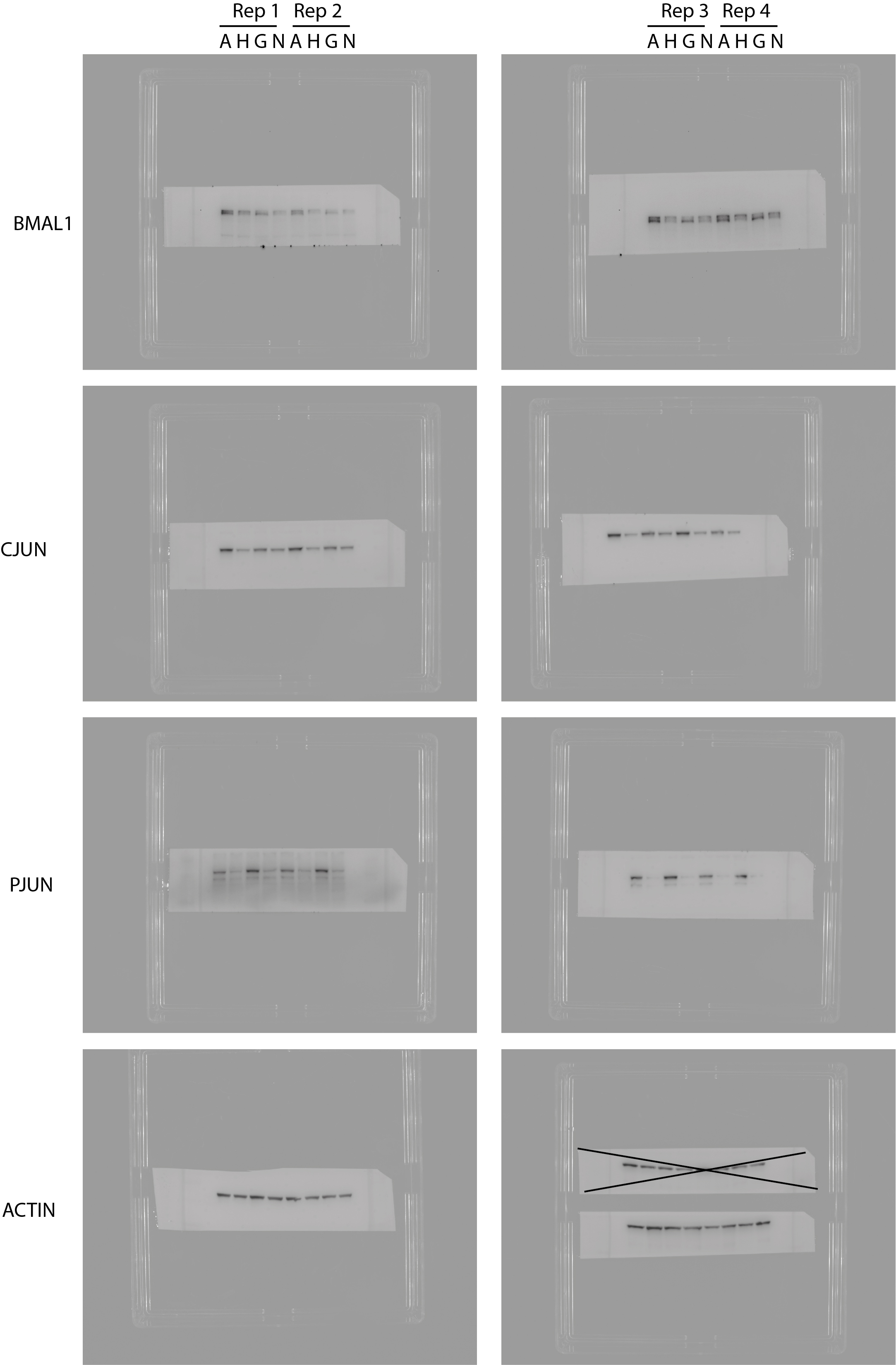

Supplement: Supplementary file 3 — Supplementary Material 3 [file 41598_2025_11617_MOESM3_ESM.jpg]
